# Cesarean Section Rates and Indications in Sub-Saharan Africa: A Multi-Country Study from Medecins sans Frontieres

**DOI:** 10.1371/journal.pone.0044484

**Published:** 2012-09-04

**Authors:** Kathryn Chu, Hilde Cortier, Fernando Maldonado, Tshiteng Mashant, Nathan Ford, Miguel Trelles

**Affiliations:** 1 Médecins sans Frontières, Johannesburg, South Africa; 2 Departments of Surgery and International Health, Johns Hopkins University, Baltimore, Maryland, United States of America; 3 Médecins sans Frontières, Medical Department, Brussels, Belgium; 4 Médecins sans Frontières, Kinshasa, Democratic Republic of Congo; 5 Médecins sans Frontières, Bujumbura, Burundi; 6 Centre for Infectious Disease Epidemiology and Research, University of Cape Town, Cape Town, South Africa; Tehran University of Medical Sciences, Iran (Republic of Islamic)

## Abstract

**Objectives:**

The World Health Organization considers Cesarean section rates of 5–15% to be the optimal range for targeted provision of this life saving intervention. However, access to safe Cesarean section in resource-limited settings is much lower, estimated at 1–2% reported in sub-Saharan Africa. This study reports Cesarean sections rates and indications in Democratic Republic of Congo, Burundi, and Sierra Leone, and describe the main parameters associated with maternal and early neonatal mortality.

**Methods:**

Women undergoing Cesarean section from August 1 2010 to January 31 2011 were included in this prospective study. Logistic regression was used to model determinants of maternal and early neonatal mortality.

**Results:**

1276 women underwent a Cesarean section, giving a frequency of 6.2% (range 4.1–16.8%). The most common indications were obstructed labor (399, 31%), poor presentation (233, 18%), previous Cesarean section (184, 14%), and fetal distress (128, 10%), uterine rupture (117, 9%) and antepartum hemorrhage (101, 8%). Parity >6 (adjusted odds ratio [aOR] = 8.6, P = 0.015), uterine rupture (aOR = 20.5; P = .010), antepartum hemorrhage (aOR = 13.1; P = .045), and pre-eclampsia/eclampsia (aOR = 42.9; P = .017) were associated with maternal death. Uterine rupture (aOR = 6.6, P<0.001), anterpartum hemorrhage (aOR = 3.6, P<0.001), and cord prolapse (aOR = 2.7, P = 0.017) were associated with early neonatal death.

**Conclusions:**

This study demonstrates that target Cesarean section rates can be achieved in sub-Saharan Africa. Identifying the common indications for Cesarean section and associations with mortality can target improvements in antenatal services and emergency obstetric care.

## Introduction

In 2008, the WHO estimated that over 350,000 women died in complications of childbirth [1]. Ninety-nine per cent of these deaths occurred in resource-poor countries [2] where access to family planning, antenatal care, and emergency obstetric services are limited. Outcome data from maternal health service data in rural Africa, where much of this mortality occurs, is difficult to obtain.

The international community, through Millennium Development Goal 5 (MDG5), has committed to reducing the maternal mortality ratio by three quarters between 1990 and 2015. In order to reach this goal, there needs to be an expansion in access to basic emergency obstetric care (EmOC) which includes the provision of antibiotics, oxytocin, and anticonvulsants, manual removal of the placenta and retained products of conception, and assisted vaginal delivery as well as comprehensive EmOC which includes safe Cesarean section and blood transfusion [3].

Cesarean section significantly reduce maternal and perinatal mortality [4]. The World Health Organization considers Cesarean section rates of 5–15% to be the optimal range for targeted provision of this life saving interventions for mother and infant [5]; lower rates suggest unmet need, while higher rates suggest improper selection. However, access to safe Cesarean section in resource-limited settings is much lower, estimated at 1–2% reported in sub-Saharan Africa. [6], [7].

No standard classification system exists for Cesarean indications [8], [9]. Cesarean sections are performed for maternal or fetal complications. One major challenge is that definitions are not standardized and indications can be multiple or related. For example, labor is considered obstructed if the presenting part of the fetus cannot enter the birth canal despite strong contractions. Cephalopelvic disproportion is the most common cause of obstructed labor. Other causes could include malpresentation (brow, transverse, or breech presentation). Labor is considered prolonged if not progressing according to a normal partogram. Different authors may classify the same patient as having obstructed labor, cephalopelvic disproportion, malpresentation or prolonged labor. Despite challenges in classification, identifying the most common indications for Cesarean section is important to target prevention strategies. In particular, recognizing indications that are associated with maternal or fetal deaths can help reduce mortality. Documentation of clinical indications in rural resource-limited settings is difficult. In particular, there is paucity of data from African maternal health services.

Medecins sans Frontieres (MSF), a humanitarian medical organization, supports emergency obstetrical programs in the Democratic Republic of Congo (DRC), Burundi, and Sierra Leone where maternal mortality ratios are extremely high, exceeding 500 deaths per 100,000 live births, compared to 9 per 100,000 live births in resource-rich countries [1].

This study reports Cesarean sections rates and indications in the DRC, Sierra Leone, and Burundi, and describe the main parameters associated with maternal and early neonatal mortality.

## Methods

### Study Setting and Sites

This study included data from four emergency obstetric programs from three sub-Saharan African countries. Cesarean sections were performed at MSF-supported or MSF hospitals. MSF-supported was defined as a government hospital which received financial and human resources support from MSF. MSF hospitals were funded, built, and staffed solely by MSF. All hospitals received patients from various clinics that provided basic emergency obstetric care. In Masisi, DRC, Masisi General Hospital served a catchement of 337,000 persons and 29 clinics. In Lubutu, DRC, Lubutu General Hospital served a catchement of 103,000 persons and 18 clinics. In Kabezi, Burundi, the Centre d'Urgence Gynéco-Obstétrique, served a catchement of 581,000 persons and 55 clinics. In Bo, Sierra Leone, Gondama Referral Centre, served a catchement of 302,000 persons and 24 clinics.

All care was provided free of charge and administered with the permission of the local health authorities. All sites had electricity, clean water, sterilization equipment, an operating theater, post-operative surgical wards, post-anesthesia care units, blood banks, anesthetics, analgesics, and antibiotics. Cesarean sections were performed by expatriate and local general surgeons, obstetricians, and general doctors with surgical skills. Anesthesia was provided by nurse anesthetists and anesthesiologists. In all four programs, MSF was the only provider of comprehensive EmOc in the catchment area although other health facilities performed vaginal deliveries and some Cesarean sections.

### Study Population

Women undergoing Cesarean section from August 1 2010 to January 31 2011 were included in this study. All Cesarean sections were performed according to peri-operative protocols established by MSF.

### Procedures

Each site had 1–3 physicians performing Cesarean sections during the study period. Each physician was trained directly by either a physician or a midwife with experience in providing CS. Definitions of each indication were included in a study guideline which was reviewed with performing physicians.

### Data Collection

Data was prospectively collected by trained data collectors using a standardized paper form and then entered into an electronic database. Regular consistency checks ensured that any missing data was tracked by the data collector and entered into the data. Baseline characteristics on age, program site, gravida, parity, and indication for cesarean section were collected. Prior to the study, various classifications of Cesarean section indications were discussed with a panel of experts with extensive experience working in sub-Saharan Africa including midwives, obstetricians and the MSF sexual health advisor. The initial indications, determined by consensus, were classified as follows: prolonged labor, pre-eclampsia/eclampsia, uterine rupture, antepartum hemorrhage (placenta previa or placental abruption), previous cesarean section, cephalopelvic disproportion, breech, malpresentation (transverse lie, face presentation, arm prolapse), multiple gestation (twins/triplets), cord prolapse, genital herpes, fetal distress, and ‘other’. For purposes of the analysis, prolonged labor and cephalopelvic disproportion were re-categorized as obstructed labor and malpresentation and breech as poor presentation. Indications for Cesarean section were classified by the physician on duty. Certain indications (such as uterine rupture) were confirmed after the procedure. Outcome data on maternal and early neonatal death were documented. Maternal death was defined as death of the mother during hospitalization. Early neonatal death was defined as death of the infant within 7 days of delivery. Cesarean section rates were calculated by dividing the total number of Cesarean section by the total number of expected deliveries. The expected number of deliveries was obtained by multiplying the crude birth rate of the country by the catchment population by the study period.

### Statistical Analysis

Baseline characteristics were described using medians and interquartile ranges (IQRs) for continuous variables and counts and percentages for categorical data. Logistic regression was used to model determinants of maternal and early neonatal mortality. Variables considered in the analysis included age, parity, program site, and indication for Cesarean section. Factors with a P<0.1 on univariate analysis were included in a multivariate model. All tests and confidence intervals were considered to be significant at a P≤0.05. All analyses were performed using STATA 10 (College Station, TX, USA).

### Ethics

Ethical approval was given by the independent Ethics Review Board of MSF. Individual patient consent was not separately obtained for the study or for patient information to be stored in the hospital database because this study was based on routinely collected data and all patient identifiers were removed prior to capture into the database and analysis.

## Results

In total 1276 women underwent a Cesarean section. The median age was 25 (IQR 20–30) years and median parity was 2 (1–5) pregnancies. 47/1276 (4%) Cesarean sections were elective. Table 1 summarizes the demographic characteristics for each program.

The most common indication was obstructed labor (399, 31%). Other indications included poor presentation (233, 18%), previous Cesarean section (184, 14%), fetal distress (128, 10%), uterine rupture (117, 9%) and antepartum hemorrhage (101, 8%) (Table 2). There were large variations between programs. Malpresentation was the most common indication in Masisi, DRC (124, 27.8%) but in Kabezi, Burundi only accounted for 11.3% (36) of cases. Previous Cesarean section was common in Kabezi (85, 26.7%) but rare in Bo, Sierra Leone (29, 8.6%). Pre-eclampsia/eclampsia accounted for 7.1% (24) of cases in Bo, Sierra Leone but less than 1% in both Masisi, DRC and Kabezi, Burundi.

**Table 1:** Demographic Characteristics of Women Undergoing Cesarean Section with Medecins sans Frontieres.

Site	Masisi	Lubutu	Bo	Kabezi	Total
n	446	174	338	318	1276
Median age (IQR)	25 (20–30)	20 (18–24)	20 (17–25)	25 (21–30)	25 (20–30)
Median parity	4 (2–7)	2 (1–4)	2 (0–4)	2 (1–4)	2 (1–5)
Emergency	415 (93)	164 (94)	333 (99)	317 (99)	1229 (96)
Median length of stay	7 (7–9)	7 (5–8)	5 (5–7)	9 (7–12)	7 (6–9)
Maternal death	2 (0.5)	0 (0)	4 (1)	1 (0.3)	7 (0.5)
Early neonatal death	54 (12)	19 (11)	72 (21)	29 (9)	174 (14)
Post-operative infection rate	32 (7)	3 (2)	25 (7)	33 (10)	93 (7)
Cesarean section rates	5.5	7.9	16.8	4.1	6.2

IQR, Interquartile range.

**Table 2:** Indications for Cesarean section.

	Masisi	Lubutu	Bo	Kabezi	Total
	n(%)	n(%)	n(%)	n(%)	n(%)
Obstructed Labor	120 (26.9)	55 (31.6)	110 (32.5)	114 (35.9)	399 (31.3)
Malpresentation	124 (27.8)	14 (8.1)	59 (17.5)	36 (11.3)	233 (18.3)
Previous Cesareansection	52 (11.7)	18 (10.3)	29 (8.6)	85 (26.7)	184 (14.4)
Fetal distress	58 (13.0)	34 (19.5)	18 (5.3)	18 (5.7)	128 (10.0)
Uterine rupture	44 (9.9)	18 (10.3)	31(9.2)	24 (7.6)	117 (9.2)
Antepartumhemorrhage	17 (3.8)	26 (14.9)	44 (13.0)	14 (4.4)	101 (7.9)
Multiple gestation	8 (1.8)	3 (1.7)	6 (1.8)	9 (2.8)	26 (2.0)
Cord prolapse	19 (4.3)	2 (1.2)	6 (1.8)	12 (3.8)	39 (3.1)
Pre-eclampsia/Eclampsia	1 (0.2)	3 (1.7)	24 (7.1)	3 (0.9)	31 (2.4)
Other	3 (0.7)	1. (0.6)	11 (3.3)	3 (0.9)	18 (1.4)
Total	446 (100)	174 (100)	338 (100)	318 (100)	1276 (100)

### Cesarean Section Rate

The Cesarean section rate was 6.2% (95% CI 11–16%), range 4.1–16.8%).

### Maternal and Early Neonatal Mortality

There were 7 (0.5%, (95% CI 0.2–1.1%)) maternal deaths. Parity greater than six (adjusted odds ratio [aOR] = 8.6, P = 0.015), uterine rupture (aOR = 20.5; P = .010), antepartum hemorrhage (aOR = 13.1; P = .045), and pre-eclampsia/eclampsia (AOR = 42.9; P = .017) were associated with maternal death. (Table 3). No maternal deaths occurred during elective Cesarean section.

**Table 3:** Associations with Maternal Mortality After Cesarean Section.

	Deaths	Univariate analysis				Multivariate analysis		
	(n/N)	%	OR	95% CI	P	aOR	95% CI	P
Age, years								
≤30	3/906	0.3	1.0 (reference)					
>30	4/370	1.1	3.3	(0.7–14.8)	0.12			
Program								
Masisi, DRC	2/446	0.4	0.74	(0.1–3.8)	0.723			
Lubutu, DRC	0/174	0	–					
Bo, Sierra Leone	4/338	1.2	3.7	(0.8–16.8)	0.086	2.4	(0.5–13.0)	0.296
Kabezi, Burundi	1/318	0.3	0.5	(0.06–4.2)	0.522			
Parity								
≤6	3/1083		1.0 (reference)					
>6	4/193		7.6	(1.7–34.3)	0.008	8.6	(1.5–48.9)	0.015
Procedure								
Elective	0/47		–					
Emergency	7/1229	0.6	–					
Indications								
Obstructed Labor	1/399	0.2	0.4	(0.4–3.0)	0.351			
Malpresentation	0/233	0	–					
Previous Cesarean section	0/184	0	–					
Fetal distress	0/128	0	–					
Uterine rupture	3/117	2.6	7.6	(1.7–34.3)	0.008	20.5	(2.1–203.8)	0.010
Antepartum hemorrhage	2/101	2.0	4.7	(0.9–24.7)	0.065	13.1	(1.1–162.5)	0.045
Multiple gestation	0/26	0	–					
Cord prolapse	0/39	0	–					
Pre-eclampsia/eclampsia	1/31	3.2	6.9	(0.8–59.0)	0.078	42.9	2.0–943.2)	0.017
Other	0/18	0	–					

OR, odds ratio; aOR, adjusted odds ratio.

There were 174 (14%, (95% CI 11–16%)) early neonatal deaths. Uterine rupture (aOR = 6.6, P<0.001), anterpartum hemorrhage (aOR = 3.6, P<0.001), and cord prolapse (aOR = 2.7, P = 0.017) were associated with early neonatal death (Table 4). These deaths were more likely to occur in the Bo, Sierra Leone program (aOR = 2.2, P<0.001). Neonates born after obstructed labor (aOR = 0.5, P = 0.004) and previous Cesarean section (aOR = 0.2, P = 0.003) were less likely die. Age <18 years of age and primigravida were not associated with mortality.

**Table 4:** Associations with Early Neonatal Mortality After Cesarean Section.

	Deaths	Unadjusted				Adjusted		
	(n/N)	%	OR	95% CI	P	aOR	95% CI	P
Age, y								
≤30	103/906	11.4	1.0 (reference)					
>30	71/370	19.1	1.8	(1.8–2.6)	<0.001	1.0	(0.6–1.6)	0.949
Program								
Masisi, DRC	54/446	12.1	0.8	(0.6–1.1)	0.244			
Lubutu, DRC	19/174	10.9	0.7	(0.5–1.2)	0.263			
Bo, Sierra Leone	72/338	21.3	2.2	(1.6–3.1)	<0.001	2.2	(1.5–3.3)	<0.001
Kabezi, Burundi	29/318	9.1	0.6	(0.4–0.9)	0.007	1.1	(0.6–1.7)	0.838
Parity								
≤6	134/1083	12.4	1.0 (reference)					
≤6	40/193	20.7	1.9	(1.3–2.7)	0.002	1.7	(1.0–2.9)	0.068
Procedure								
Elective	3/47	6.4	1.0 (reference)					
Emergency	171/1229	13.9	2.4	(0.7–7.7)	0.152			
Indications								
Obstructed Labor	22/399	5.5	0.3	(0.2–0.4)	<0.001	0.5	(0.3–0.8)	0.004
Malpresentation	27/233	11.6	0.8	(0.5–1.2)	0.314			
Previous Cesarean section	5/184	2.7	0.2	(0.06–0.4)	<0.001	0.2	(0.09–0.6)	0.003
Fetal distress	16/128	12.5	0.9	(0.5–1.6)	0.693			
Uterine rupture	53/117	45.3	7.1	(4.7–10.7)	<0.001	6.6	(4.1–10.6)	<0.001
Antepartum hemorrhage	35/101	34.7	4.0	(2.6–6.2)	<0.001	3.6	(2.2–6.2)	<0.001
Multiple gestation	2/26	7.7	0.5	(0.1–2.2)	0.380			
Cord prolapse	9/39	23.1	1.9	(0.9–4.2)	0.086	2.7	(1.2–6.1)	0.017
Pre-eclampsia/eclampsia	3/31	9.7	0.7	(0.7–2.2)	0.518			
Other	2/18	11.1	0.8	(0.2–3.5)	0.754			

OR, odds ratio; aOR, adjusted odds ratio.

## Discussion

In this study, obstructed labor and poor presentation accounted for half of Cesarean sections. These findings are consistent with a systematic review that identified these as leading indications for Cesarean [10]. While in this study these indications were not associated with maternal or neonatal mortality, it is estimated that 8% of maternal deaths worldwide are from obstructed labor, the majority of these deaths occurring in Africa [11]. Obstructed labor can also lead to other complications such as uterine rupture, fetal distress/death and obstetric fistulae.

Previous Cesarean section accounted for 14% of Cesarean sections, although this rate varied by program, accounting for almost a quarter of Cesarean sections in Kabezi, Burundi. Safety of childbirth after Cesarean section is a public health concern [12] because the risk of uterine rupture is higher. If women in Africa who undergo Cesareans are at risk of potentially unnecessary future Cesarean sections, they must be counseled about the risks of complications.

Uterine rupture accounted for 10% for Cesarean sections. Antepartum hemorrhage accounted for approximately 8% of Cesarean sections and was as high as 14% in some sites. Both indications were associated with maternal and early neonatal demise. These maternal complications must be identified early so that Cesarean sections can be performed in a timely manner.

Grand multiparity was a risk factor for maternal mortality in this study. This is consistent with other studies from sub-Saharan Africa. [13], [14] Increased access to family planning and reducing the unmet need for contraception would reduce mortality in this group.

Prevention of labor complications includes general improvement of women’s health such as nutrition status and the delay of childbirth. Reproductive health programs must expand coverage of antenatal care. Women at risk for complications must be identified prior to or in early labor and systems for referral must be expanded.

### Early Neonatal Mortality

One in seven Cesarean sections resulted in early neonatal death. This high rate may reflect inadequate labor management and the late transfer of patients from home or a health center to the hospital. In Kabezi, Burundi, where the early neonatal death rate was only 9%, a well-organized system of referral was in place. 20 health centers in the province are equipped with radios and three ambulances are available 24 hours a day, 7 days a week to transfer patients needing comprehensive EmOC including Cesarean sections. In contrast, the early neonatal death rate at the in Bo, Sierra Leone was 21%, a setting in which many patients from non-MSF supported clinics and outside the catchement area utilized the hospital; these patients may not have received adequate prenatal and early labor management. Early neonatal death rate was only 12% when referred from MSF-supported clinics. This points to the need for improved basic EmOC and referral resources at all health clinics.

### Cesarean Section Rate

Although the mean Cesarean section rate in this study (6.2%) was well within the WHOs recommended range, this rate is not representative of all regions of the DRC, Burundi, and Sierra Leone. MSF was the main provider of comprehensive EmOC in the study catchment areas, performing over 90% of Cesarean sections. It must be assumed that the Cesarean section rate in other areas is much lower, as access to the procedure is more limited.

Strengths of this study include the fact that it is based on systematic, prospective reporting of Cesarean section rate and indications, and associations with maternal and early neonatal mortality. There are also a number of potential limitations. It is possible that Cesarean section rates were overestimated since vaginal deliveries at the home and at non-health facilities may have been underreported. It is less likely that the number of Cesarean sections were underreported since few non-MSF health facilities provided Cesarean sections. The death rates in our study were likely underestimated as while WHO defines maternal death as any death within 42 days of delivery, our study defined maternal death as death during hospitalization. Similarly, the neonatal death rate may have been underestimated since they were only captured during hospitalization.

In conclusion, our report from four projects in three of the most impoverished countries in sub-Saharan Africa demonstrates that target Cesarean section rates and low maternal and neonatal mortality can be achieved with support of non-governmental agencies. Complications can be lowered with better antenatal services and basic EmOC at the health clinic level [15], [16]. In order to move closer to the MDG5 target, the international community should continue to support the local health authorities to ensure that sufficient knowledge and resources are available to make safe motherhood a sustainable goal.
